# Preclinical evaluation of multi antigenic HCV DNA vaccine for the prevention of Hepatitis C virus infection

**DOI:** 10.1038/srep43531

**Published:** 2017-03-07

**Authors:** Hyojin Lee, Moonsup Jeong, Jooyeon Oh, Youngran Cho, Xuefei Shen, John Stone, Jian Yan, Zachary Rothkopf, Amir S. Khan, Byung Mun Cho, Young K. Park, David B. Weiner, Woo-Chan Son, Joel N. Maslow

**Affiliations:** 1 GeneOne Life Science, Seoul, Korea; 2 Department of Pathology, University of Ulsan College of Medicine, Asan Medical Center, Seoul, Korea; 3 Inovio Pharmaceuticals, Plymouth Meeting, PA, USA; 4 Department of Medicine, Morristown Medical Center, Morristown, NJ, USA; 5 Wistar Institute, Philadelphia, PA, USA

## Abstract

Direct-acting antiviral treatment for hepatitis C virus (HCV) infection is costly and does not protect from re-infection. For human and chimpanzees, recovery from acute HCV infection correlates with host CD4+ and CD8+ T cell responses. DNA plasmids targeting the HCV non-structural antigens NS3, NS4, and NS5, were previously reported to induce robust and sustained T cell responses in mice and primates. These plasmids were combined with a plasmid encoding cytokine IL-28B, together named as VGX-6150. The dose-dependent T cell response and safety of VGX-6150 administered intramuscularly and followed by electroporation was assessed in mice. Immune responses plateaued at 20 μg/dose with IL-28B demonstrating significant immunoadjuvant activity. Mice administered VGX-6150 at 40, 400, and 800 μg given either as a single injection or as 14 injections given bi-weekly over 26 weeks showed no vaccine related changes in any clinical parameter compared to placebo recipients. There was no evidence of VGX-6150 accumulation at the injection site or in any organ 1 month following the 14^th^ vaccination. Based on these studies, the approximate lethal dose (ALD) exceeds 800 μg/dose and the NOAEL was 800 μg/dose in mouse. In conclusion, VGX-6150 appears safe and a promising preventive vaccine candidate for HCV infection.

Hepatitis C virus (HCV) was discovered in 1989 and is recognized as the major cause of transfusion and community acquired non-A and non-B hepatitis^1^. More than 170 million people, 2.2~3.0% of the world’s population, are chronically infected with HCV^2^,^3^. Although spontaneous clearance of infection has been documented, at least 70% of HCV infections result in chronic illness^4^. Despite reductions in viral transmissions related to transfusion from effective screening of blood donors, the trend has been offset by a sharp increase of injection drug use among adolescents and young adults and concomitant spread of HIV^5^,^6^. Treatment of hepatitis C virus infection had initially relied on interferon-based regimens which were associated with significant side effects, commonly requiring dose reduction, and cure rates of 60% only for limited subgroups of patients. While newer directly acting antiviral agents (DAAs) are well tolerated and have cure rates of 90–95%, the high cost has resulted in less than 10% of those infected being treated in resource-rich nations. Resource poor nations with limited health-care budgets are extremely constrained as to the ability to offer treatment. Moreover, treatment does not protect against re-infection. Therefore, effective strategies to block the transmission of HCV globally are still needed.

The possibility of inducing protective immune responses against hepatitis C to prevent infection is suggested by the immunology of spontaneous clearance of infection. Spontaneous resolution of HCV infection, observed in 30% of cases, is thought to protect against re-infection^7^. Studies of hepatitis C virus infection in chimpanzees showed HCV infected animals have much shorter duration and peak magnitude of viremia after re-challenge with virus even years later^8^. Studies have shown potential correlates of protective immunity against Hepatitis C infection. The role of sterilizing immunity is questioned because people with chronic infection manifest broadly neutralizing responses yet may still develop re-infection following re-exposure^4^. In contrast, clearance of acute HCV infection in humans and chimpanzees is correlated with T cell responses^9^,^10^. Clearance of viremia is associated with the onset of virus-specific T cell immunity, whereas failure to mount T cell responses is regarded as a reliable predictor of persistent viremia. Long-lived memory CD4+ and CD8+ T cell responses after resolution of acute infection are also rapidly recalled after re-infection of virus in human and chimpanzees^11^,^12^,^13^. Therefore, an ideal HCV vaccine to prevent viral persistence should induce strong, broad and polyfunctional CD4+ and CD8+ T cell responses and sustained memory T cell responses.

DNA vaccines designed by SynCon^®^ technology delivered by electroporation are safe, stable, quickly produced, well tolerated, and induce robust, broad and poly-functional T cell immune responses^14^. A recent clinical Phase IIb clinical trial has shown that a DNA vaccine targeting the human papilloma virus genotype 16 and 18 E6 and E7 proteins was able to generate robust antigen-specific CD8+ responses with T-cell reactivity directly correlating to clinical response in the treatment of high grade cervical neoplasia^15^. The cytokines IL-12 and IL-28 have recently gained attention as possible adjuvants for DNA vaccines^16^. IL-12 directly stimulates the cytotoxic ability of CD8+ cells by increasing the production of IFN-γ and TNF-α^17^. IL-28B markedly increases antigen-specific IFN-γ, inducing T Helper 1 (Th1) cells which aid CD8+ cell functionality^18^. Tolerance of CD8+ cells, defined by impaired cytotoxicity and decreased IFN-γ production is observed in chronic Hepatitis C viremia. Therefore, reversal of the CD8+ tolerant state in chronic hepatitis C patients could ameliorate the course of viremia.

Kuhs *et al*. previously reported on the development of a DNA vaccine against hepatitis C virus that encoded for the HCV NS3/4A, NS4B, and NS5A proteins as target antigens^19^,^20^. For clinical development, IL-28B was selected as a DNA plasmid-based immune adjuvant because of its action through the IFNγ pathways and its induction of Th1 immunity. This HCV DNA vaccine product named VGX-6150 is comprised of 4 plasmids, 3 plasmids encoding for the non-structural target antigens and 1 plasmid encoding for the IL-28B adjuvant. Here we report on the preclinical assessment of VGX-6150. First, we evaluated T cell immune responses of mixed, broad ranged antigens compared with each antigen alone and second, we evaluated the immune adjuvant function of plasmid expressing IL-28B. Then, we confirmed the safety profile of VGX-6150 in both GLP animal toxicology and biodistribution studies.

## Results

### Rationale for toxicology dose and animal

Results of previous preclinical and clinical studies using DNA vaccines cloned into the same plasmid backbone delivered by electroporation demonstrated a high safety profile in animals and humans^21^,^22^. In this toxicology and biodistribution study, the dosage of DNA vaccine was increased to supra-therapeutic doses and a second study examined a dose regimen that far exceeded the number of vaccinations that would be used in clinical practice. Human doses of plasmid DNA for vaccination have generally ranged between 0.1 mg to 6 mg/dose with a few studies up to 10 mg/dose. In Korea, toxicology assessments for DNA-based products have required evaluation in one or more animal species, including rodents. C57BL/6 mice were selected as the study animal. For mice, a dose of 40 μg would correspond to a dose of 10 mg for humans based on allometric dosing principles. Dosing levels were therefore chosen as 40 μg/dose as low dose, 400 μg/dose (10 fold greater than a typical dose in human trials), and 800 μg/dose corresponding to a dose 20-fold greater than the typical dose used in human clinical trials.

### Single dose intramuscular toxicology and biodistribution study of VGX-6150 in C57BL/6 mice

Toxicology and biodistribution studies were conducted at the Korea Institute of Toxicology (Daejeon, Korea) under GLP conditions. To investigate the acute toxicity of VGX-6150, C57BL/6 mice were assigned to 4 groups, vehicle control (WFI, water for injection), T1 (40 μg/dose), T2 (400 μg/dose), and T3 (800 μg/dose), with 12 animals/both sexes/group and vaccinated with VGX-6150 intramuscularly with electroporation (EP). Clinical assessments detected no changes in body weight, other clinical parameters, or evidence of mortality. Histopathology of tissue taken at necropsy 15 days following vaccine administration was deferred as all clinical assessments were normal. Therefore the approximate lethal dose (ALD) of VGX-6150 was considered to be greater than 800 μg/animal for both male and female mice.

**Figure 1:**
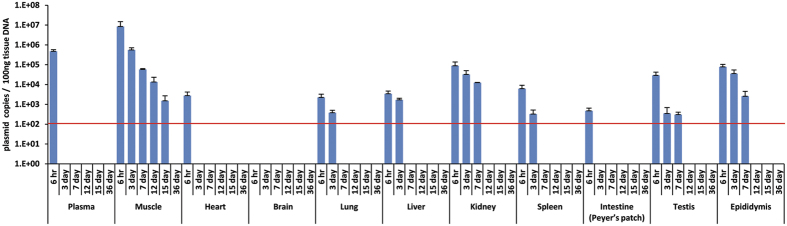
Biodistribution of VGX-6150 after single immunization. C57BL/6 mice were injected with 800 μg of VGX-6150 intramuscularly. Several organs were collected at several time points, 6 hr, 3 day, 7 day, 12 day, 15 day and 36 day. The amount of VGX-6150 in organs was detected with qPCR (LOQ = 100 copies/100 ng total DNA). The red line indicates the lower limit of detection.

Biodistribution following a single dose of VGX-6150 was assessed in organs and blood at several time points: 6 hr, 3 day, 7 day, 12 day, 15 day, and 36 day. Following injection at a dose of 800 μg, with a calculated amount of plasmid administered as 1.7 × 10^14^ copies, AUC in blood was measured as 1.4 × 10^5^ copies X day/mL, Cmax is 1.1 × 10^6^ copies/mL, and T_1/2_ is 3.2 hours (Table 1). If we consider the volume of whole blood in mice as 3 mL, we can infer the maximal amount of VGX-6150 as 3.3 × 10^6^ copies in blood, which is about 1 × 10^−6^% of the calculated copy number of injected product, likely representing a small amount of leakage from muscle at the immunization site. Persistence of VGX-6150 was also assessed in several organs (Fig. 1). VGX-6150 was detected at high levels in muscle at the site of injection immediately following injection but dissipated over 15~36 days. Dissemination to other organs was low due to rapid degradation of VGX-6150 in blood. The lack of accumulation of VGX-6150 in reproductive organs, testis and epididymis at 12 days is informative to reproductive concerns related chromosomal DNA integration and reproductive toxicity.

### 26-week intramuscular repeated dose toxicity study of VGX-6150 in C57BL/6 mice with 4-week and 8-week recovery periods

**Figure 2:**
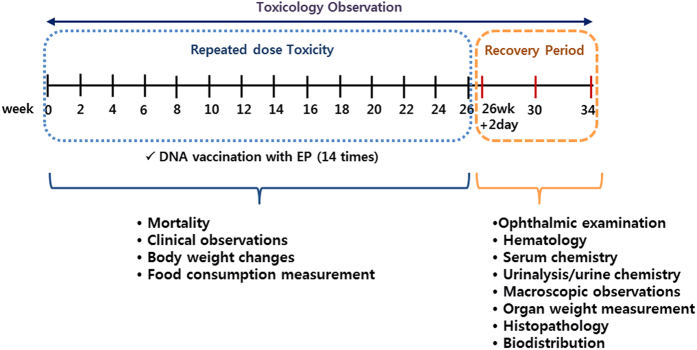
Scheme of repeated dose toxicity study for VGX-6150. C57BL/6 mice were divided into 3 dosing groups, 40 μg, 400 μg, and 800 μg, n = 24 per group, and injected with VGX-6150 intramuscularly, 14 times, 2 weeks interval. Toxicology was evaluated by GLP toxicology CRO, KIT.

**Figure 3:**
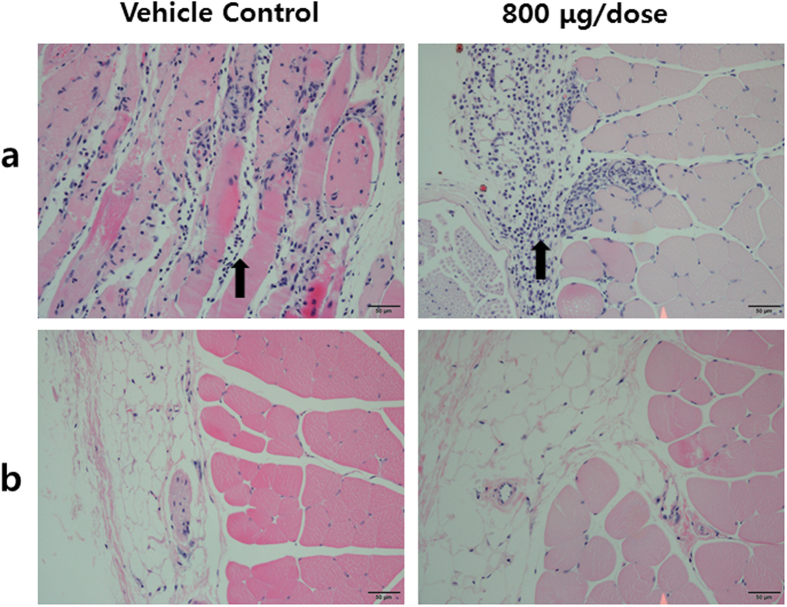
Representative histopathological findings in injection sites. In 2 days after 14 vaccinations, the infiltration of mixed inflammatory cells (black arrow) were observed in epimysium or perimysium of injection sites in control or treated groups (**a**). In recovery period of 4-weeks, the lesions were not seen (**b**). Bar = 50 μm.

Typical vaccination schedules for DNA vaccines in humans employ between 3 and 4 doses administered over time, ranging from 1–4 dose administrations. For VGX-6150, human dose schedules were expected to be include at least 3 or 4 vaccinations. C57BL/6 mice were assigned to 4 groups, vehicle control (WFI), T1 (40 μg/dose), T2 (400 μg/dose), and T3 (800 μg/dose), with 12 animals/both sexes/group and vaccinated with VGX-6150 intramuscularly with electroporation biweekly for 26 weeks, for a total of 14 vaccinations, and followed by 4 or 8-week recovery periods. Therefore, the “typical” schedule of vaccination was far exceeded as part of this study. The protocol mandated that should any clinical or laboratory abnormalities be observed, additional assessments were conducted at 29 days or 34 days later until the abnormality resolved. Toxicology assessments included mortality, general clinical observation, body weight changes, food consumption measurement throughout study period and ophthalmic examination as well as hematology, serum chemistry, urinalysis/urine chemistry, macroscopic observations, organ weight measurement, and histopathology at 2 days after 26 week study. The schema of the study is depicted in Fig. 2. Biodistribution of VGX-6150 was assessed at 2 or 3 days and at 29 days after 26 week study period (Fig. 2). There were no VGX-6150 related changes in mortality, clinical observation, body weight, food consumption, ophthalmic examination, urine chemistry, hematology, and organ weight compared to animals injected with WFI. An inflammatory mixed cell infiltration was observed in the injection site of both sexes at 0(WFI), 40, 400 and 800 μg/dose and in skeletal muscle epimysium of both sexes at 800 μg/dose, but disappeared after recovery period of 4- week (Tables 2 and 3, Fig. 3). In 800 μg/dose group, VGX-6150 was injected into two separate injection sites in contralateral hindlimbs due to limitation in the 10 mg/mL formulation of VGX-6150. The mixed cell inflammatory infiltrate was considered to have occurred as a direct result from the volume and number of injections into skeletal muscle of both sexes at 800 μg/dose group. The group mean aspartate aminotransferase (AST) was significantly higher in both sexes at 800 μg/dose at 2–3 days following the final injection but was not different from placebo at 29 days; this change was not considered as clinically relevant (Table 4). The changes were thus considered by the assessing veterinarians as non-adverse effects since the magnitude of change was small and were fully recovered after recovery periods of 4-week or 8-week. Therefore, the NOAEL (No Observed Adverse Effect Level) is considered to be at least 800 μg/dose in both sexes in this study.

**Figure 4:**
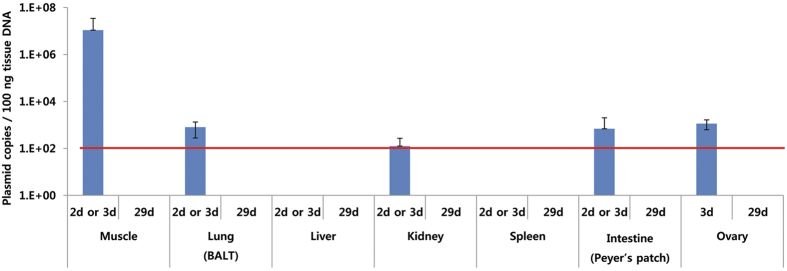
Biodistribution of VGX-6150 after 14 immunizations. C57BL/6 mice were injected with 800 μg of VGX-6150 intramuscularly, 14 times, 2 weeks interval. Several organs were collected at two time points, 2 or 3 day and week 4 after 26 week study. The amount of VGX-6150 in organs was detected with qPCR (LOQ = 100 copies/100 ng total DNA).

The biodistribution of VGX-6150 after 14 bi-weekly injections was also assessed on day 2 or 3 and at 4 weeks following the final injection (Fig. 4). At 2–3 days, the level of VGX-6150 in tissue was consistent with its decay from the primary injection site. There was no evidence of accumulation of VGX-6150 in the muscle at the injection site or in any organ 1 month following the 14^th^ injection.

### 12-week intramuscular repeated dose lymphocytes analysis study of VGX-6150 in C57BL/6 mice with 4-week and 8-week recovery period

**Figure 5:**
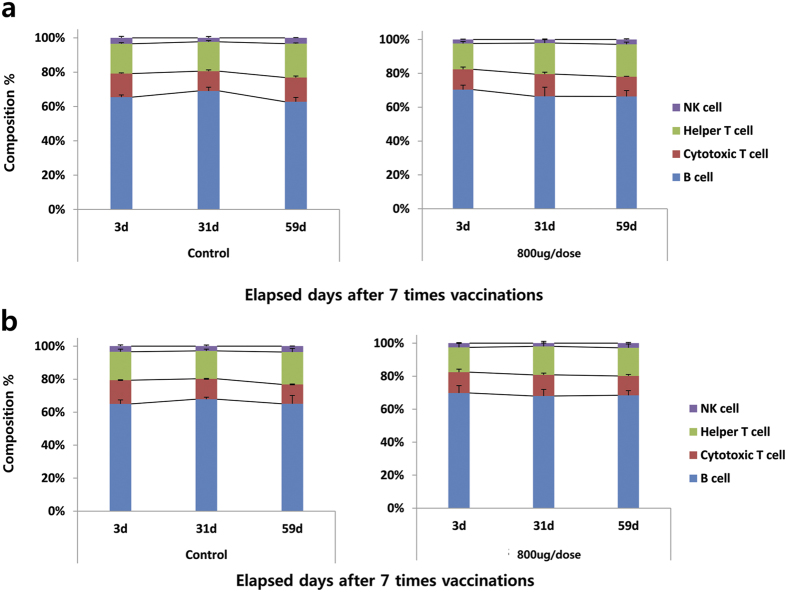
Lymphocyte analysis after 7 times immunizations of VGX-6150. C57BL/6 mice, 6 mice/sexes/group, were injected with 800 μg of VGX-6150 intramuscularly, 7 times, 2 weeks interval. The numbers of lymphocytes per microliter were measured on day3, day 31, and day 59 after 12 week study in male group (**a**) and female group (**b**).

To evaluate whether multiple vaccinations of VGX-6150 can induce alterations in splenic lymphocyte subsets, C57BL/6 mice were assigned to 4 groups, WFI (vehicle control), T1 (40 μg/dose), T2 (400 μg/dose), and T3 (800 μg/dose), with 12 animals/both sexes/group and vaccinated with VGX-6150 intramuscularly with electroporation biweekly for 12 weeks (total 7 times), and in order to observe the reversibility of changes over 4-week and 8-week after 12 week study period, WFI control and T3 group included additional animals with 6 animals/both sexes/group and those additional groups were named as recovery groups. During the study, there was no VGX-6150 related clinical sign and body weight change. Analysis of splenic lymphocytes was conducted at day 3, day 31, and day 59 after 12 week study. There were no significant changes in NK cells, B cells, Helper T cells and cytotoxic T cells in mice as part of either study (Fig. 5). In conclusion, the repeated intramuscular vaccinations of VGX-6150 to C57BL/6 mice for 12 weeks including 8 week recovery period showed no VGX-6150-related changes in splenic lymphocytes.

### VGX-6150 induces HCV-specific, dose-dependent, T cell response

**Figure 6:**
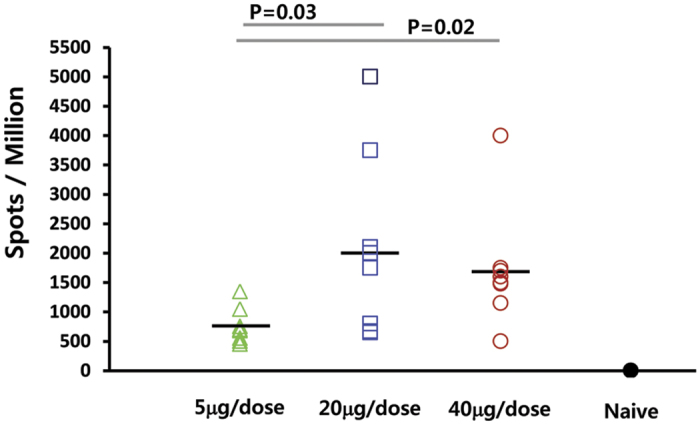
VGX-6150 induces HCV-specific T cell response in a dose-dependent manner. Balb/c mice were divided into 3 dosing groups, 5 μg, 20 μg, and 40 μg, n = 24 per group, and immunized with VGX-6150 intramuscularly, 3 times, 2 weeks interval. The number of NS3/4A, NS4B, and NS5A specific IFN-γ spots per million splenocytes was determined through IFN-γ ELISpot assays.

In order to determine the dose dependency of HCV specific T cell immune response induced by VGX-6150, Balb/c mice (n = 8) were immunized intramuscularly 3 times at 2 week intervals with three different doses of VGX-6150, 5 μg, 20 μg, and 40 μg. Peptide pools spanning the NS3/4A, NS4B, and NS5A proteins were the same as used in previous studies^19^,^20^,^23^. As shown in Fig. 6, VGX-6150 induced a potent T cell immune response that was showed an increased response between 5 and 20 μg with saturation of responses above 20 μg/dose. Therefore, based on these data, allometric principles would predict that the estimated human dose for saturation of T-cell responses would be 5 mg/dose.

### Comparison of IL-28B adjuvanted T cell immune responses of VGX-6150 versus NS3/4A immunization alone

**Figure 7:**
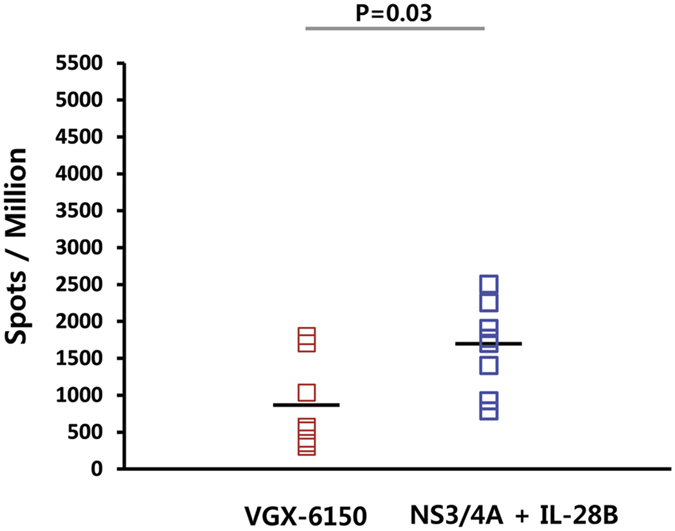
VGX-6150 induces enhanced and broad T cell responses. Balb/c mice were immunized with 20 μg of VGX-6150 or with plasmids pGX8005 (NS3/4A) and pGX6014 (IL-28B) intramuscularly, 3 times, 2 weeks interval. The numbers of NS3/4A specific IFN-γ spots per million splenocytes were determined through IFN-γ ELISpot assays.

Next, we studied if combination of vaccine plasmids encoding different antigens can affect the induction of T cell responses by a single antigen, i.e. whether a multi-antigen vaccine would result in interference. Balb/c mice (n = 8) were immunized intramuscularly with 20 μg/dose followed by EP, 3 times at 2 weeks interval. T-cell responsiveness was determined by IFN-γ ELIspot assay. VGX-6150 at 20 μg, which contains 6 μg each of the three plasmids encoding for NS3/NS4A, NS4B, and NS5A and 2 μg of IL-28B, induced lower NS3/4A specific T cell responses than 6 μg NS3/4A alone given together with 2 μg of IL-28B plasmid +12 μg backbone plasmid with a p value of 0.03 (Fig. 7). This difference was considered as clinically within normal variation and in the context of multiple antigens should not adversely affect overall immune response

### IL-28B produced from VGX-6150 acts as effective immune adjuvant

In order to determine the function of IL-28B as an immune adjuvant, VGX-6150 was compared to the same plasmid formulation but without IL-28B. Balb/c mice (n = 8) were immunized intramuscularly with 20 μg/dose followed by EP, 3 times, at 2 weeks interval, and IFN-γ ELIspot assays performed to measure T-cell responses.

**Figure 8:**
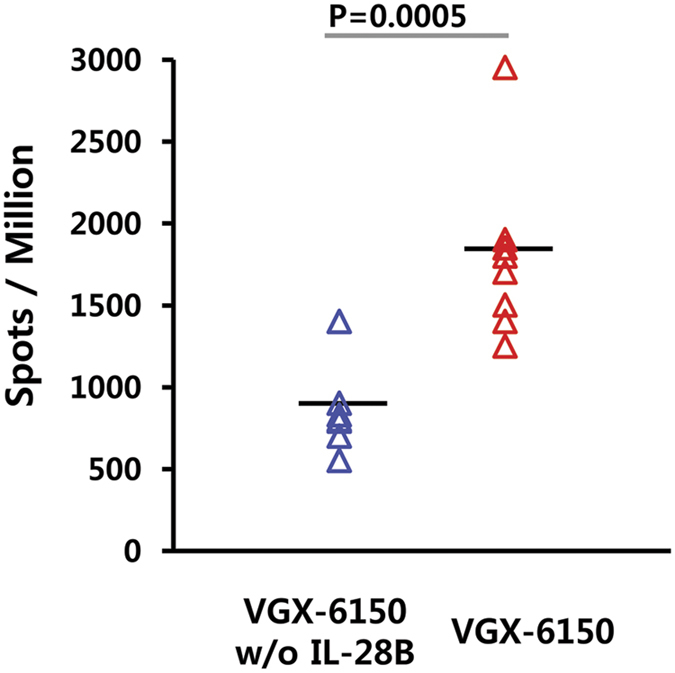
IL-28B produced from VGX-6150 acts as effective immune adjuvant. Balb/c mice were immunized with 16 μg of VGX-6150 or with plasmids pGX8005 (NS3/4A), pGX8006 (NS4B), and pGX8007 (NS5A) without the addition of pGX6014 (IL-28B) intramuscularly, 3 times, 2 weeks interval. The number of NS3/4A, NS4B, and NS5A specific IFN-γ spots per million splenocytes was determined through IFN-γ ELISpot assays.

As shown in Fig. 8, VGX-6150 induced T cell immune responses twice as great as VGX-6150 w/o mIL-28B. This demonstrates a potent adjuvant function of IL-28B.

## Discussion

The recent advent of highly effective direct-acting antiviral (DAA) treatment with 90–95% cure rates, improved tolerability and a comparably short duration, up to 12 weeks, has increased optimism about achieving eradication of HCV^24^,^25^,^26^. However, therapy with DAAs has not been widely accepted internationally, including within resource rich countries, due to their cost. Moreover, DAA regimens do not provide protection from re-infection or serve as prophylaxis among individuals at high-risk for incident infection. Thus, an effective vaccine to prevent HCV re-infection would still provide a significant benefit to the overall treatment of hepatitis C virus infection^27^.

Until now, two vaccines targeting hepatitis C have been developed for clinical use. One vaccine developed by Chiron (now Novartis) uses recombinant envelope glycoproteins E1 and E2 as antigenic targets to induce neutralizing antibodies^28^. At present, the relative contribution of cellular immune response versus neutralizing antibodies in protection from persistence cannot be determined. No follow-up trials of this vaccine have been described. The other, developed by Okairos (now GlaxoSmithKline), uses the expression of HCV non-structural proteins from recombinant viruses to induce CD8+ T cell immunity^29^. The vaccine regimen involving priming with a chimpanzee adenovirus (serotype chAd3) and boosting with modified vaccinia virus Ankara was recently evaluated^30^. When compared with the heterologous adenovirus boost used in their first clinical study, MVA boosting provided a very substantial increase in CD4+ and CD8+ T cell responses and broadening of the response to non-structural proteins of multiple HCV genotypes^30^. A Phase I/II clinical trial of the latter vaccine is now underway in individuals at risk for HCV infection due to injection drug use (ClinicalTrials.gov NCT01436357). To prevent viral persistence effectively, strong T cell responses including CD4+ and CD8+ T cells and prolonged memory T cell response against HCV are considered as necessary because sterilizing immunity is not protective in chronic hepatitis C. For human use, recombinant viral vaccines, such as the MVA construct above, require careful assessment for adverse events; they are limited in use in pregnancy and cannot be used in immunosuppressed individuals due to the possibility of disseminated infection. Additionally, for some viral vaccines neutralizing antibodies against the viral backbone that may either limit immune reactivity, and has even been associated with increased risk for disease^31^. Therefore, to induce strong T cell responses and to overcome the drawbacks of vaccination using recombinant virus, we applied DNA vaccine technology to develop first-in-class preventive HCV vaccine.

We assessed a DNA vaccine targeting the HCV non-structural antigens, NS3, NS4, and NS5, and including plasmid expressing IL-28B as immune adjuvant, which were mixed in one formulation and named as VGX-6150. First, our vaccine elicited T cell responses in mice which plateaued at 20 μg/dose. This may relate to a saturation of immune responses in humans at approximately 5 mg/dose as inferred by allometric conversion between mice and humans. Interestingly, when we compared T cell responses induced by single antigen with mixed 3 antigens, the response to NS3/NS4 was lower when mice were vaccinated with the combined product VGX-6150 versus an equivalent dose of the single plasmid given with IL-28B. This would suggest the possibility of interference between plasmids, or could represent competition between plasmids for antigen production within cells. These findings would need to be confirmed in larger mammal models and requires further study. Whether this difference is clinically significant or not is unknown as the threshold for protective responses is unknown. Prior vaccine candidates with similar construction induced robust polyfunctional T cell responses and sustain memory T cell response^19^,^22^. Taken together, our vaccine could be a most promising preventive vaccine for HCV.

We used the plasmid expressing IL-28B as an adjuvant for the HCV DNA vaccine. Our vaccine will be the first vaccine to use DNA based IL-28B as adjuvant in human. In this study, we demonstrated enhanced T cell responses by the addition of IL-28B in mice. IL-12 has also been used as an immunoadjuvant, however, one different aspect of IL-12 is that will bias towards a Th1 type immune response, IFN-γ release, with increased cytotoxicity and may have a short-lived effect^16^,^17^,^18^. After this period, cytokine levels and cell populations could possibly return to baseline although this has not been observed in the clinic where IL-12 increased T cell responses for at least 6 months^32^. IL-28B may be unique in the HCV setting as IL-28B gene is directly linked to the ability of an HCV infected patient to control and clear HCV infection^33^,^34^,^35^. There are three genotypes of IL-28B in humans, CC, CT and TT. The IL-28B CC genotype is associated with spontaneous HCV viral clearance. Additionally patients who do not spontaneously clear but who are more likely to respond to PEG-IFN and RBV also express the CC genotype. These and other studies suggest that lower levels of IL-28B are associated with a uniquely altered immune response which plays an important role in HCV responses. Accordingly, we reasoned that IL-28B is a unique genetic adjuvant that may have advantages for immune therapy in HCV patients, thus VGX-6150 has the potential to elicit long lasting immune responses and could reverse CD8+ tolerance in HCV patients.

This study assessed the toxicology and biodistribution in mice of VGX-6150, which contains three separate plasmids that encode for hepatitis C non-structural proteins, as well as the immune adjuvant IL-28B that was also plasmid based. All 4 plasmids were based on a common backbone, pGX0001, a modified pVAX1 expression system. The study assessed doses that would be typical for human clinical use (40 μg/dose) as well as doses considered well above this level (400 μg and 800 μg/dose). Moreover, while most vaccination schedules utilize 2 to 4 doses given over time, this study assessed the toxicology and biodistribution of both repeated vaccination with 14 doses of vaccine administered at 2 week intervals over a period of 26 weeks. In these studies, there was no lethality observed at any administered dose and the NOAEL was 800 μg/dose in mouse. If we convert 800 μg/dose of mouse into a human equivalent dose, the corresponding dose would be 240 mg/dose which is significantly higher than an average dose for DNA vaccine in human which ranges from 1 to 10 mg/dose. At 4 weeks following repeated injection, VGX-6150 in injection site muscle was not detectable. Additionally, this is the first toxicology and biodistribution assessment of the immunoadjuvant IL-28B for which no adverse effects were observed in mice. As a further test of the toxicology of IL-28B, it was observed that there were no changes in splenic lymphocytes after 7 immunizations.

During chronic infection, CD4+ T cells are difficult to detect in blood or liver, and during persistent HCV infection, CD8+ T cells are functionally exhausted. This is likely to be due to negative regulation involving inhibitory signaling through receptors like PD-1 and regulatory T cell activity^11^. Although DAA have a high rate of cure for chronic hepatitis C, there is no protection from re-infection and is a concern raised to long-term control of HCV transmission^7^. One longitudinal study of two patients before and after cure showed the increased CD127 expression on CD8+ T cells targeting an invariant epitope and PD-1 expression decreased slightly, suggesting at least partial recovery of circulating CD8+ T cells from exhaustion^36^. In fact, a Phase I clinical trial showed that inhibition of PD-1 blockade with BMS-936558 (now approved as Opdivo^®^) can result in temporary suppression of HCV replication in some patients with chronic infection, including those who do not respond to interferon-alfa therapy^37^. It suggests that further studies of PD-1 pathway blockade may be beneficial, possibly in combination with other immunomodulatory agent including our HCV DNA vaccine or direct-acting antiviral agents.

Based on these preclinical results and the aforementioned benefits of VGX-6150, we are conducting a Phase I clinical study to evaluate the safety and immunogenicity of VGX-6150 in Korea (ClinicalTrials.gov identifier: NCT02027116). Thus, VGX-6150 appears to be a potent immunogen and may have a role in the treatment and/or prevention of hepatitis C infection.

## Materials and Methods

### Generation of VGX-6150

The generation of plasmids expressing NS3/4A, NS4B, NS5A, and murine IL-28B, designated as pGX8005, pGX8006, pGX8007, and pGX6014, respectively, have been previously described^16^,^19^,^23^. The sequence for murine IL-28B was obtained from NCBI GenBank Database, and was synthesized, codon optimized, and subsequently subcloned into the modified pVAX1 backbone, pGX0001, by GenArt (Renensberg, Germany). VGX-6150 was comprised of pGX8005, pGX8006, pGX8007, and pGX6014 at a mass ratio of 3:3:3:1. VGX-6150 used for the toxicology study was produced under non-GMP conditions by VGXi, Inc. (The Woodlands, TX).

### Immunization and Electroporation

Six to ten week old female Balb/c mice were purchased from Charles River Laboratories (Wilmington, MA). The mice were immunized on week 0, 2, and on week 4 (for a total of 3 doses). All animal housing and experimentation were conducted in accordance with the guidelines set by NIH. DNA vaccine was delivered by intramuscular (IM) injection into the quadriceps femoris muscle of defined amounts of plasmid in 30 μl sterile water and followed immediately by electroporation. Electroporation was applied to the site of injection using CELLECTRA^®^ adaptive constant current device (Inovio Pharmaceuticals Inc., Plymouth Meeting, PA). Electroporation was delivered using a program of 2 sets of 2 pulses (2 × 2), with an interval between pulses of 0.2 seconds, and an interval of 3 seconds between the two sets of pulses and with pulse duration of 52 milliseconds. Electrical parameters were programed with a constant current of 0.2 Amp, and maximal voltage of 200 V based on the impedance within the muscle which was measured dynamically during the electroporation procedure.

### Splenocyte isolation

Spleens were isolated and individually crushed with the use of a cell strainer and sterile plunger. Splenocytes were strained through a 40 mM cell strainer and treated 5 min with ACK lysis buffer (Thermo Fisher Scientific, Waltahm, MA) to lyse RBCs. The splenocytes were resuspended in complete media (Rosewell Park Memorial Institute (RPMI) 1640 with 2 mM/L L-glutamine supplemented with 10% heat inactivated FBS, 10 mM HEPES, 0.1 mM MEM non-essential amino acid, 1 mM MEM sodium pyruvate, 1x anti-biotic/anti-mycotic, and 0.05 mM/L ß-mercaptoethanol).

### IFN-γ ELISpot

The mouse IFN-γ ELISpot assay was performed by using Mouse IFN-γ Development Module (R&D Systems, Minneapolis, MN) following the manufacturer’s instructions. Splenocytes were added in triplicate at an input cell number of 2 × 10^5^ cells/well resuspended in RPMI medium 1640 with 10% fetal bovine serum. Splenocytes were stimulated with pools of 15mer peptides overlapping by 8 amino acids and spanning the length of each antigen. Peptides of NS3/4A, NS4B, and NS5A were synthesized by GenScript (Piscataway, NJ), resuspended in DMSO and pooled at a concentration of 1 mg/ml/peptide. Results were adjusted and graphed as the average number of spot forming units (SFU) per 1 × 10^6^ splenocytes. Concavalin A (Sigma-Aldrich, St. Louis MO), at 4 mg/ml, was used as a positive control. The color development was performed according to the manufacturer’s instructions (ELISPOT Blue Color Module, R&D Systems, Minneapolis, MN). The spots on the plates were counted using an automated ELISPOT reader system.

### HCV NS3/NS4A, NS4B, and NS5A peptide sequence

Peptides spanning the NS3/4A, NS4B, and NS5B regions were synthesized as 15-mer oligomers overlapped by 8 residues (Invitrogen, Thermo Fisher Scientific, Waltham, MA). Each peptide was dissolved in DMSO at 20 mg/ml first and then combined to pools. Approximately 20 peptides were combined into a peptide pool as shown in Table 5.

### Toxicology

Toxicity studies were conducted under Good Laboratory Procedures, Ministry of Food and Drug Safety (Good Laboratory Practice Regulations for Non-clinical Laboratory Studies, Notification No. 2013-40) at the Korea Institute of Toxicology (Daejeon, Korea) in compliance with the Test Guidelines for Safety Evaluation of Drugs, Ministry of Food and Drug Safety (Test Guidelines for Safety Evaluation of Drugs: Annex 2. Repeated Dose Toxicity Study, Notification No. 2013-121). Experiments were reviewed and approved by KIT- Institutional Animal Care and Use Committee (No. 1211-0340). Six to seven week old male and female C57BL/6 mice were purchased from Orient Bio Inc. (Gyeonggi-Do, Korea). Groups of mice were immunized with VGX-6150 either once or 14 times at 2 weeks interval with either 40, 400 and 800 μg of VGX-6150 in 50 μl water administered IM into the quadriceps femoris and followed immediately by *in vivo* electroporation. Clinical assessments were performed twice daily during the treatment period. Body weights and food consumption were measured once at animal receipt and before grouping, respectively, and at 3~7 day intervals during the treatment period. Ophthalmological examinations were performed on all animals once during the acclimation period and again during the final week of treatment. Ophthalmological examinations were performed using fundus camera (IO-H, Neitz Instrument Co., Japan or Vantage Plus Digital, Keeler LTD., England) and slit lamp (SL-5, Kowa Co. Ltd., Japan) after pre-treatment with mydriatic (Mydrin-P, Santen Pharmaceutical Co., Japan). Since no VGX-6150-related changes were observed, ophthalmological examination was not performed during the recovery period. The hematological parameters evaluated in this study included total leukocyte count (WBC), total red blood cell count (RBC), hemoglobin (HGB), hematocrit (HCT) and mean corpuscular volume (MCV). The serum chemistry parameters included glucose (GLU), blood urea nitrogen (BUN), creatinine (CREA), total protein (TP), albumin (ALB), albumin/globulin ratio (A/G), total cholesterol. The urinalysis/urine chemistry parameters were color (COL)/clarity (CLA), pH, bilirubin (BIL), protein and nitrite. Mice were sacrificed either at the final immunization or specified time points, and were euthanized by isoflurane inhalation. Organs were collected and weighed at necropsy to include the liver, spleen, heart, thyroid glands, kidneys, thymus, lungs, brain, adrenal glands, seminal vesicles, epididymis, ovaries, testes, prostate, uterus, pituitary gland, and salivary gland. Tissues were preserved in 10% neutral buffered formalin, except for the eyes (including the optic nerve) which were fixed in Davidson’s fixative and the testes (including the epididymis) which were fixed in Bouin’s fixative.

### Biodistribution

Biodistribution study was conducted under GLP at the Korea Institute of Toxicology. Dosing of VGX-6150 and immunization methods were the same as for the toxicity studies. Experiments were reviewed and approved by KIT- Institutional Animal Care and Use Committee (No. 1208-0275). Biodistribution in blood and tissue was determined after a single immunization of VGX-6150 followed by electroporation was assessed at 6 hr, day 3, day 7, day 12, day 15 and day 36 following vaccination. To evaluate biodistribution for animals immunized 14 times with VGX-6150, samples were collected either at day 2 or 3 following the final immunization or at week 4. The organs assessed in the biodistribution studies included the ovaries, testis, epididymis, intestine, lung, spleen, kidney, liver, brain, heart, blood, and muscle (the injection site), which were collected and then stored at −80 °C until analysis. Total DNA was separated from tissue using QIAamp DNA Mini Kit (Qiagen Korea Ltd, Seoul, Korea). VGX-6150 was detected and analyzed quantitatively by performing real-time qPCR with Taqman probes. The detection region for qPCR is Kanamycin resistant gene, selection maker for plasmid, which is present in all plasmids in VGX-6150. The primers and the base sequence of the probe used in this experiment are as follows: Primer (F): 5′-GGCGCCCGGTTCTTTT-3′, Primer (R): 5′-GCGCTGCCTCGTCTTG-3′, Probe: 5′-FAM-CCGGTGCCCTGAATGA-3′. After 10 min of AmpliTaq gold activation at 95 °C, the samples were amplified for 40 cycles of denaturation at 95 °C for 15 sec, annealing/extension at 60 °C for 1 min a ABi 7500 Q-PCR system (Applied Biosystems, Thermo Fisher Scientific). The limit of quantitation was 100 plasmid copies/100 ng total DNA. After qPCR, quantitation of VGX-6150 (plasmid copy number) was calculated using ABi 7500 software. The amplification cycle value (Ct value) at fluorescence signal level (threshold) with statistical significance was applied to the linearized standard curve for quantitative analysis, and the results, obtained by repeating the process twice for each sample, were averaged. To verify the validity of the test method, the assay values satisfied the following criteria: the correlation coefficient of the obtained standard curve ≥0.980, assay value of the negative control < limit of quantification.

### Statistics

Spots/million splenocytes for each individual mouse and the mean of each group of mice were calculated. The statistical difference between a pair of immunized groups was assessed using a two-tailed unpaired t test that generated a specific P-value. Two groups with a P-value < 0.05 were considered to be statistically different and therefore significant.

## Additional Information

**How to cite this article:** Lee, H. *et al*. Preclinical evaluation of multi antigenic HCV DNA vaccine for the prevention of Hepatitis C virus infection. *Sci. Rep.* **7**, 43531; doi: 10.1038/srep43531 (2017).

**Publisher's note:** Springer Nature remains neutral with regard to jurisdictional claims in published maps and institutional affiliations.

## Figures and Tables

**Table 1:** PK parameters of VGX-6150 in serum after single immunization.

Parameter	Value
AUC (copies X day/mL)	1.4 × 10^5^
Cmax (copies/mL)	1.1 × 10^6^
T_1/2_ (hour)	3.2

*C57BL/6 mice were injected with 800 μg of VGX-6150 intramuscularly. Blood was collected at several time points, 6 hr, 3 day, 7 day, 12 day, 15 day and 36 day. The amount of VGX-6150 in blood was detected with qPCR (LOQ = 100 copies/100 ng total DNA).

**Table 2:** Histopathological findings of repeated dose toxicity study.

Controls from group(s): 1	Animal sex	Males				Females			
Dosing units: μg/dose	Group dosage level	0	40	400	800	0	40	400	800
Tissues with diagnoses	No. in group	12	12	12	12	12	12	12	12
SKELETAL MUSCLE	Number examined	12	12	12	12	12	12	12	12
Inflammatory cell infiltration, mixed cells, epimysium		0	0	0	5*	0	0	0	5*
INJECTION SITE	Number examined	12	12	12	12	12	12	12	12
Inflammatory cell infiltration, mixed cells, epimysium		0	4	3	2	0	6*	3	7*
Inflammatory cell infiltration, mixed cells, perimysium/endomysium		1	1	2	1	0	4	1	2

Incidence summary table with statistics for 2 or 3 days following 14 immunizations.

Note: Entries flagged with a 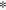 are significantly different from control at the 0.05 level using Fisher’s exact two tailed test. In 800 μg/dose group, VGX-6150 was injected on both legs due to limitation to make formulation of VGX-6150 having over 10 mg/mL. All Diagnoses; Phases: P3; Death types: All scheduled; Date of death range: 16-Apr-13 To 17-Apr-13.

**Table 3:** Histopathological findings of repeated dose toxicity study.

Controls from group(s): 1	Animal sex	Males		Females	
Dosing units: μg/dose	Group dosage level	0	800	0	800
Tissues with diagnoses	No. in group	6	6	6	6
SKELETAL MUSCLE	Number examined	6	6	6	6
Inflammatory cell infiltration, mixed cells, epimysium		0	0	0	0
INJECTION SITE	Number examined	6	6	6	6
Inflammatory cell infiltration, mixed cells, epimysium		0	0	0	0
Inflammatory cell infiltration, mixed cells, perimysium/endomysium		0	0	0	0

Incidence summary table with statistics for 4 weeks following 14 immunizations.

Note: Entries flagged with a 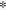 are significantly different from control at the 0.05 level using Fisher’s exact two tailed test. All Diagnoses; Phases: P4; Death types: Scheduled S1; Date of death range: 14-May-13 To 14-May-13.

**Table 4:** Average level of aspartate aminotransferase in serum during repeated dose toxicity study.

Elapsed days after 14 times immunizations	2 or 3 days				4 weeks				8 weeks			
	Male(n = 12)		Female(n = 12)		Male(n = 6)		Female(n = 6)		Male(n = 6)		Female(n = 6)	
Group dosage level (μg/dose)	Mean	SD	Mean	SD	Mean	SD	Mean	SD	Mean	SD	Mean	SD
0	70.5	28.6	49.9	8.33	56.6	4.5	464.5	9.49	53.3	3.68	65.2	3.82
40	92.1	49.35	55.4	10.7	—	—	—	—	—	—	—	—
400	71.1+	24.88	55.6	7.75	—	—	—	—	—	—	—	—
800	115*	35.53	70.7	13.7	53.7	3.34	63.8	6.64	56.8	4.02	63.4	6.45

^+^Significant difference from control, 0 μg/dose, group (p < 0.05).

^*^Significant difference from control, 0 μg/dose, group (p < 0.01).

**Table 5:** Strategy for making peptide pool of each antigen.

Peptide pool	Pool1	Pool2	Pool3	Pool4	Pool5
NS3/4A	Peptides 1–20	Peptides 21–40	Peptides 41–60	Peptides 61–80	Peptides 81–101
NS4B	Peptides 1–18	Peptides 19–37			
NS5A	Peptides 1–21	Peptides 22–42	Peptides 43–64		
